# Supplementary material for: Sugar-Linked Diethyldithiocarbamate Derivatives: A Novel Class of Anticancer Agents
Source: Int J Mol Sci. 2025 Jun 11;26(12):5589. doi: 10.3390/ijms26125589 (PMC12193388; doi:10.3390/ijms26125589)
Supplement: Supplementary file 1 [file ijms-26-05589-s001.zip › ijms-3642466-supplementary.pdf]

International Journal of Molecular Sciences

MDPI

Supplementary Materials

Sugar-Linked Diethyldithiocarbamate Derivatives: A Novel Class of Anticancer Agents

Mohammad Najlah *, Niamh McCallum, Ana Maria Pereira, Dan Alves, Niusha Ansari-Fard, Sahrish Rehmani and Ayşe Kaya

Pharmaceutical Research Group, School of Allied Health, Faculty of Health, Education, Medicine and Social Care, Anglia Ruskin University, Bishops Hall Lane, Chelmsford CM1 1SQ, UK
* Correspondence: mohammad.najlah@aru.ac.uk

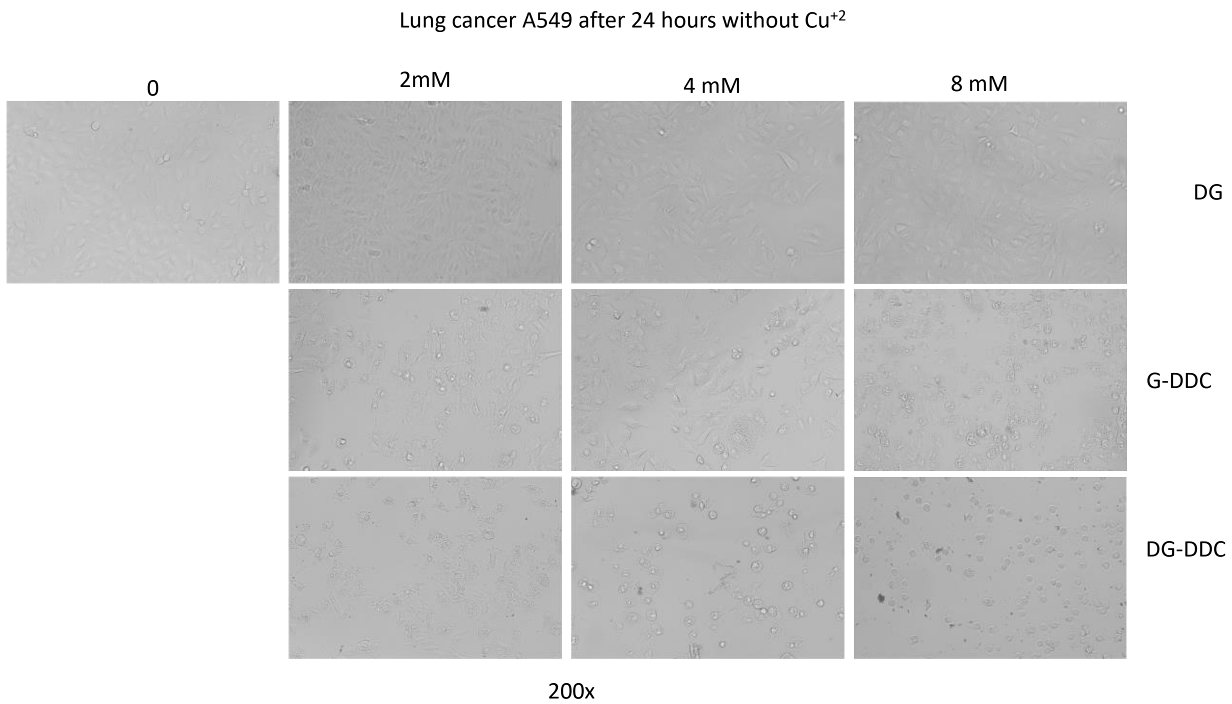

Figure S1 shows microscopy images of lung cancer cell line A549 at 200x magnification in 0 mM, 2 mM, 4 mM, 8 mM concentrations of glycosyl diethyldithiocarbamate (G-DDC), 2-deoxy-glycosyl diethyldithiocarbamate (DG-DDC) and 2-deoxy-glucose (DG), all without copper(II) after 24 hours.

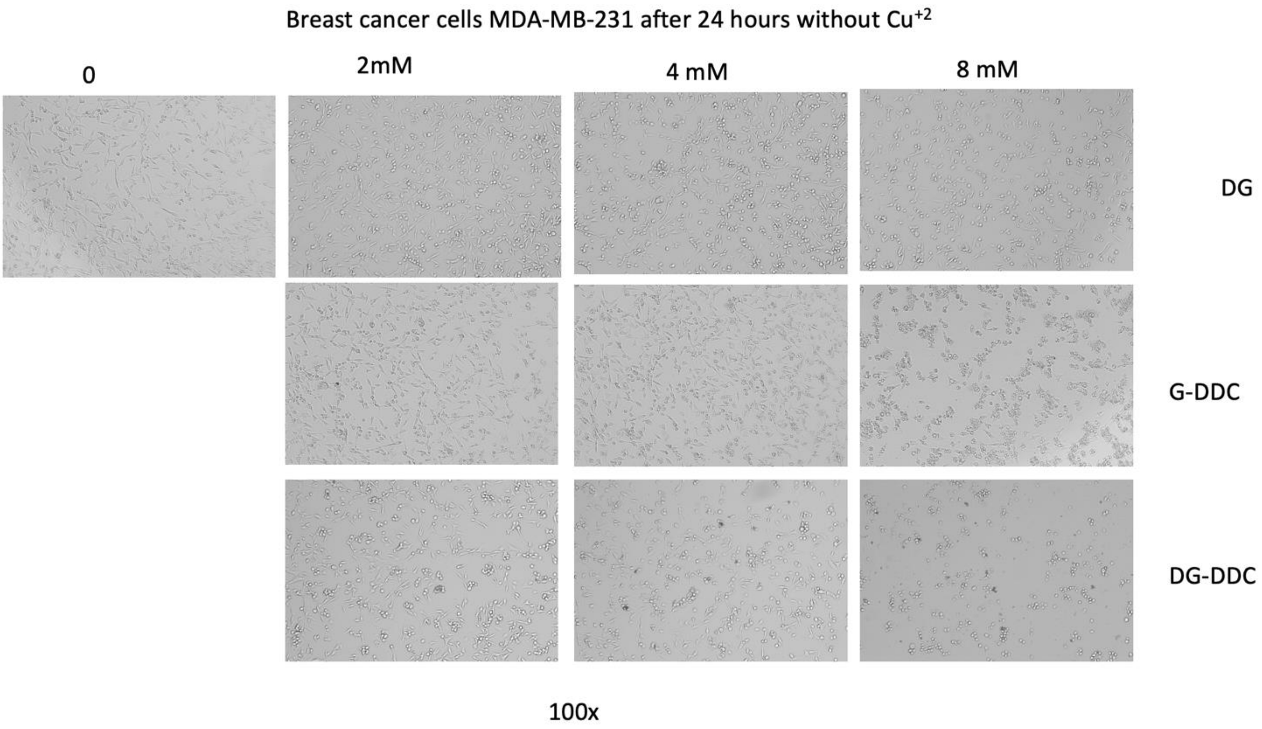

Figure S2 shows microscopy images of breast cancer cell line MDA-MB-231 at 100x magnification in glycosyl diethyldithiocarbamate (G-DDC), 2-deoxy-glycosyl diethyldithiocarbamate (DG-DDC) and 2-deoxy-glucose (DG) after 24 hours, all without copper(II).

Breast cancer cells MDA-MB-231 after 24 hours with Cu^{2+} (10 μM)

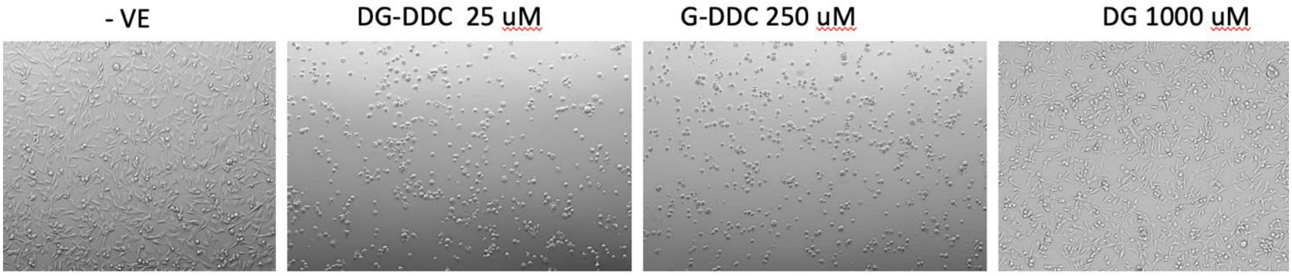

Figure S3 shows microscopy images of breast cancer cell line MDA-MB-231 at 100x magnification in 25 μM 2-deoxy-glycosyl diethyldithiocarbamate (DG-DDC), 250 μM glycosyl diethyldithiocarbamate (G-DDC), and 1,000 μM 2-deoxy-glucose (DG) after 24 hours, all with 10 μM copper(II).

Lung cancer cells A539 72 hours of incubation (100x)

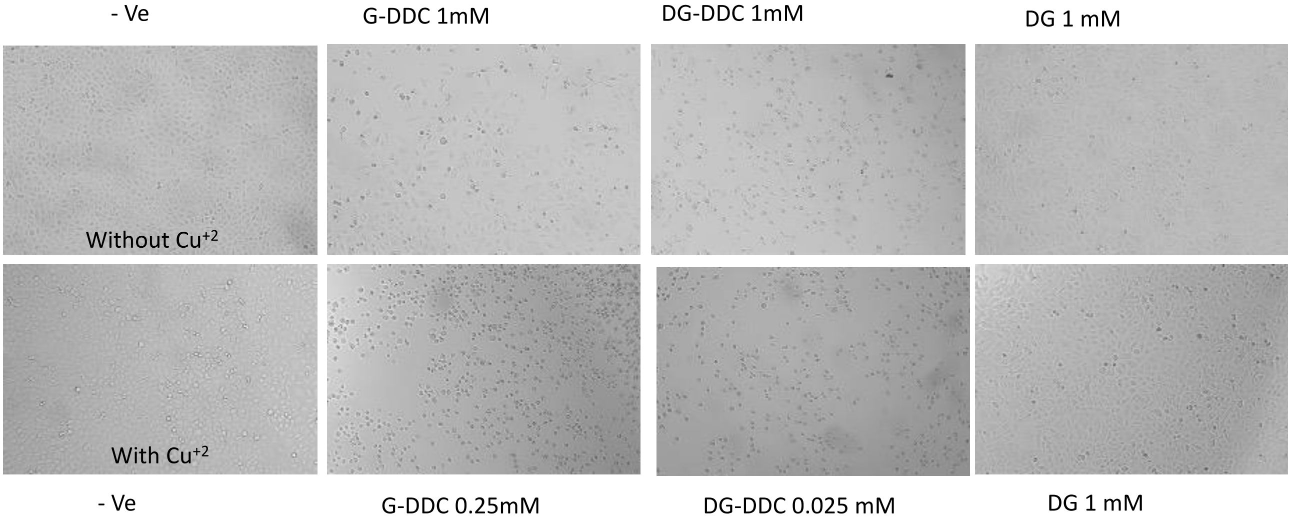

Figure S4 shows microscopy images of lung cancer cell line A549 at 100x magnification in 1 mM glycosyl diethyldithiocarbamate (G-DDC), 1 mM 2-deoxy-glycosyl diethyldithiocarbamate (DG-DDC) and 1 mM 2-deoxy-glucose (DG) after 72 hours, without copper(II), and 0.25 mM glycosyl diethyldithiocarbamate (G-DDC), 0.025 mM 2-deoxy-glycosyl diethyldithiocarbamate (DG-DDC) and 1 mM 2-deoxy-glucose (DG) after 72 hours, with copper(II).

Colorectal cancer cells H630 WT after 72 hours with Cu^{2+} (10 μM)

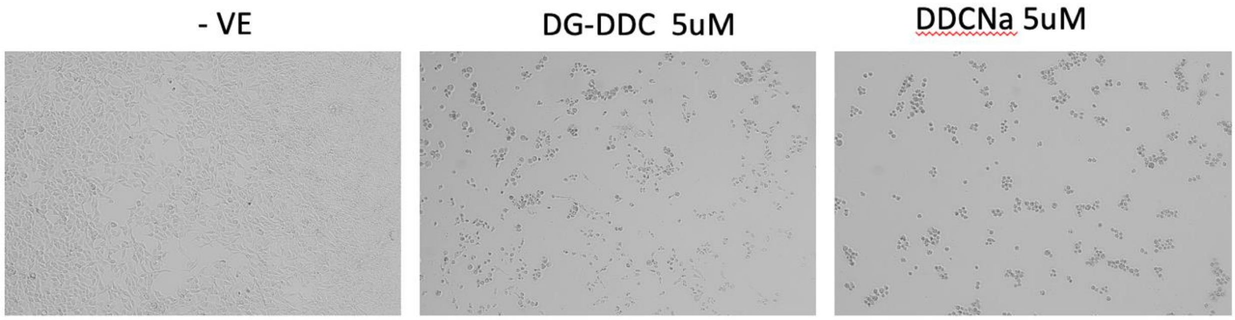

Figure S5 shows microscopy images of colorectal cancer cell line H630 WT at 100x magnification in 2-deoxy-glycosyl diethyldithiocarbamate (DG-DDC) and sodium diethyldithiocarbamate (DDC-Na) after 72 hours, all with 10 μM copper(II).

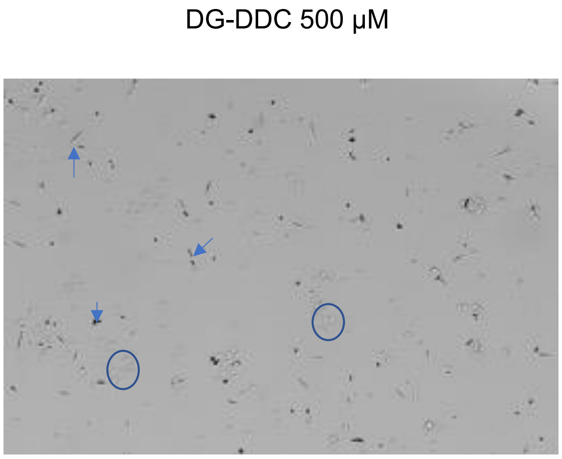

Figure S6 shows microscopy images of colorectal cancer cell line H630 WT at 100x magnification in 2-deoxy-glycosyl diethyldithiocarbamate (DG-DDC) 500 μM all with 10 μM copper(II), arrows indicate $Cu(DDC)_2$ crystals and cycles show dead cells.

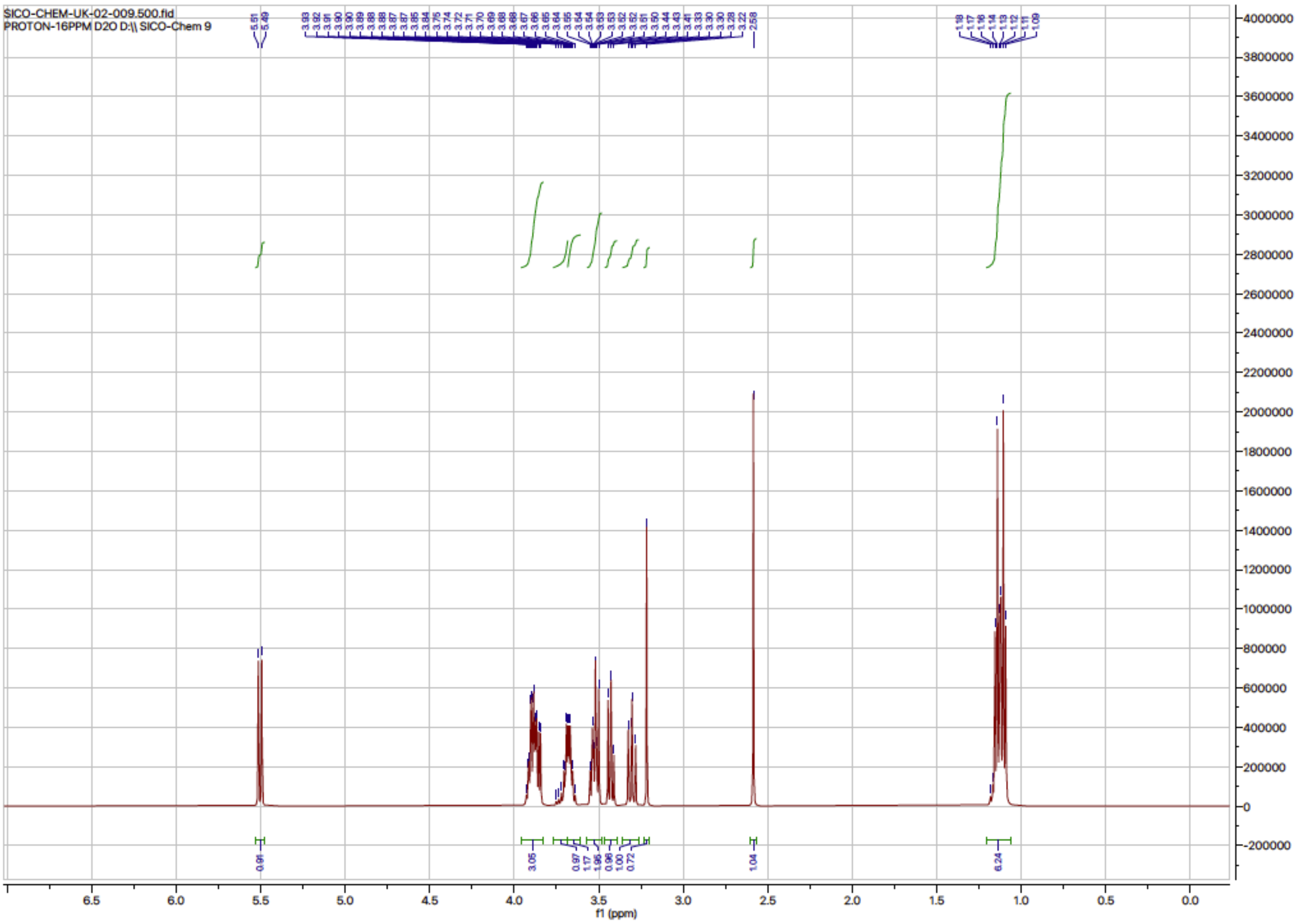

Figure S7. ^{1}H NMR of XY-DDC

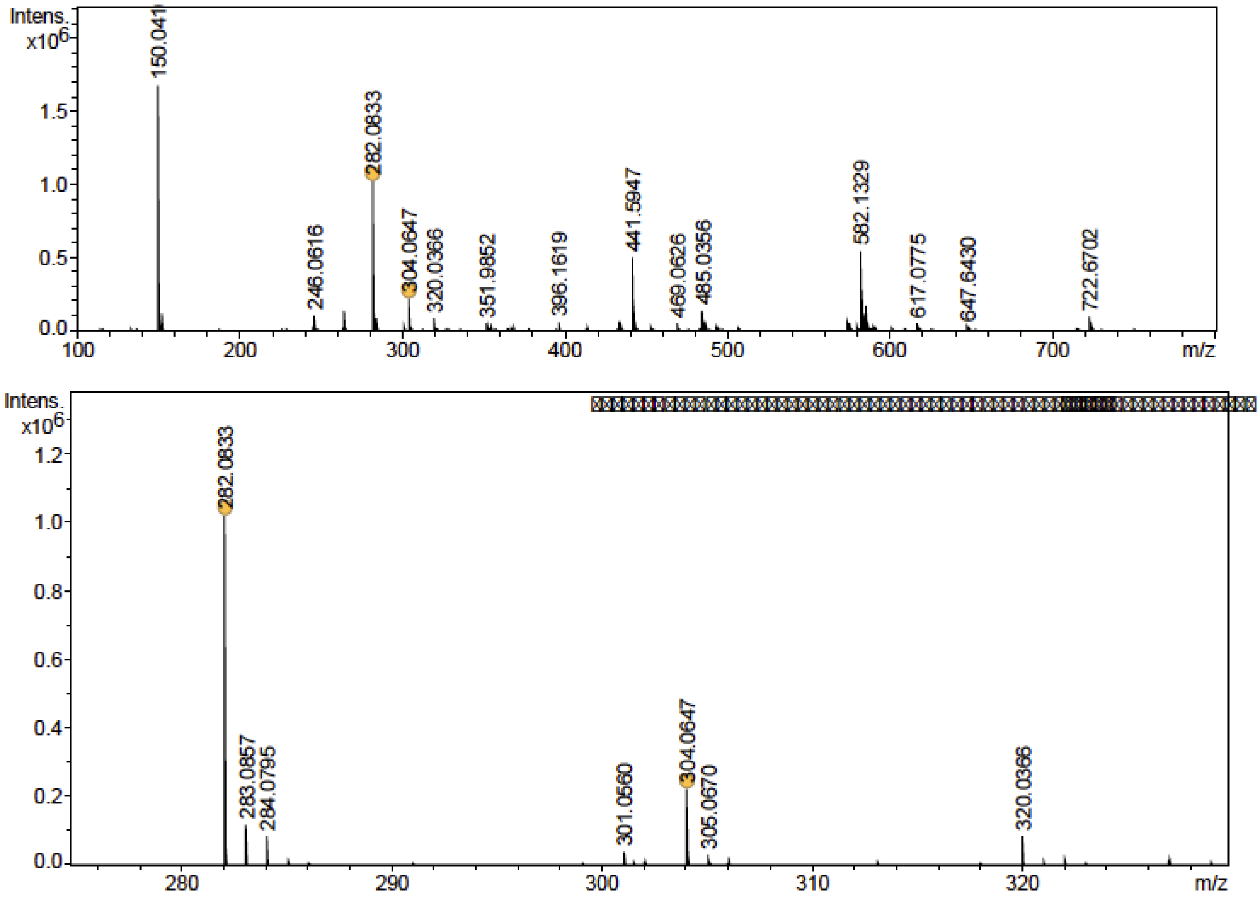

Figure S8. MS-ESI of XY-DDC (M+1 = 282, M+Na = 304)
